# Supplementary material for: The wtf4 meiotic driver utilizes controlled protein aggregation to generate selective cell death
Source: eLife. 2020 Oct 27;9:e55694. doi: 10.7554/eLife.55694 (PMC7591262; doi:10.7554/eLife.55694)
Supplement: Transparent reporting form [file elife-55694-transrepform.pdf]

1st Floor
24 Hills Road
Cambridge CB2 1JP, UK

P 01223 855340
W elifesciences.org
T @elife

eLife's transparent reporting form

We encourage authors to provide detailed information *within their submission* to facilitate the interpretation and replication of experiments. If you have any questions, please contact us: editorial@elifesciences.org.

Sample-size estimation

- You should state whether an appropriate sample size was computed when the study was being designed
- You should state the statistical method of sample size computation and any required assumptions
- If no explicit power analysis was used, you should describe how you decided what sample (replicate) size (number) to use

Please outline where this information can be found within the submission (e.g., page numbers or figure legends), or explain why this information doesn't apply to your submission:

We did not use power analyses to determine the sample sizes we assayed. Working with microbes, it is straightforward to assay many cells for each phenotype. Our general approach was to assay >20 cells for each quantified cell biological assay. For the phenotypes that were not quantified, we observed hundreds of cells and imaged and presented representative cells.

The sample sizes we used for the following experiments are listed on the figures themselves and/or in the materials and methods section on the indicated page:

1. Pearson Correlation (Figure 1-figure supplement 1C, Figure1-figure supplement 2, Figure1-figure supplement 3C and 3E)
2. Nuclear size (Figure 1-figure supplement 4B, Figure 2-figure supplement 1D)
3. FRET (Figure 2F, Figure 5C), page 33.
4. half-FRAP (Figure 5B, Figure 5-figure supplement 3B), page 33
5. Organelle quantification (Figure 4D, Figure 4-figure supplement 2F-G)
6. *S. pombe* fluorescence microscopy, page 30 and for *S. cerevisiae* fluorescence microscopy, page 31.
7. Electron microscopy, page 34.
8. Allele transmission (Figure1-figure supplement 3A)

Replicates

- You should report how often each experiment was performed
- You should include a definition of biological versus technical replication
- The data obtained should be provided and sufficient information should be provided to indicate the number of independent biological and/or technical replicates
- If you encountered any outliers, you should describe how these were handled
- Criteria for exclusion/inclusion of data should be clearly stated

eLife Sciences Publications, Ltd is a limited liability non-profit non-stock corporation incorporated in the State of Delaware, USA, with company number 5030732, and is registered in the UK with company number FC030576 and branch number BR015634 at the address 1st Floor, 24 Hills Road, Cambridge CB2 1JP | August 2014

eLIFE
1st Floor
24 Hills Road
Cambridge CB2 1JP, UK
P 01223 855340
W elifesciences.org
T @elife

- High-throughput sequence data should be uploaded before submission, with a private link for reviewers provided (these are available from both GEO and ArrayExpress)

Please outline where this information can be found within the submission (e.g., page numbers or figure legends), or explain why this information doesn't apply to your submission:

We assayed at least two independent (biological and technical replicates) diploids in each gametogenesis experiment.
We assayed at least three independent cultures (biological and technical replicates) in each vegetative growth experiment.

Statistical reporting

- Statistical analysis methods should be described and justified
- Raw data should be presented in figures whenever informative to do so (typically when N per group is less than 10)
- For each experiment, you should identify the statistical tests used, exact values of N, definitions of center, methods of multiple test correction, and dispersion and precision measures (e.g., mean, median, SD, SEM, confidence intervals; and, for the major substantive results, a measure of effect size (e.g., Pearson's r, Cohen's d)
- Report exact p-values wherever possible alongside the summary statistics and 95% confidence intervals. These should be reported for all key questions and not only when the p-value is less than 0.05.

Please outline where this information can be found within the submission (e.g., page numbers or figure legends), or explain why this information doesn't apply to your submission:

We used t-tests and highlighted values where the p was less than 0.01, although they were all much smaller. This can be found in figure legends of (Figure1-figure supplement 4B, Figure2-figure supplement1D, Figure4D, Figure 5C).

We also used a g-test and highlighted the value where the p was less than 0.01 in the gametogenesis experiment. This can be found in figure legend of (Figure1- figure supplement 3).

(For large datasets, or papers with a very large number of statistical tests, you may upload a single table file with tests, Ns, etc., with reference to page numbers in the manuscript.)

Additional data files ("source data")

- We encourage you to upload relevant additional data files, such as numerical data that are represented as a graph in a figure, or as a summary table
- Where provided, these should be in the most useful format, and they can be uploaded as "Source data" files linked to a main figure or table
- Include model definition files including the full list of parameters used

- Include code used for data analysis (e.g., R, MatLab)
- Avoid stating that data files are "available upon request"

Please indicate the figures or tables for which source data files have been provided:

Figure5 has source data (Figure5-source data1)
